# Rabies molecular virology, diagnosis, prevention and treatment

**DOI:** 10.1186/1743-422X-9-50

**Published:** 2012-02-21

**Authors:** Muhammad Zubair Yousaf, Muhammad Qasim, Sadia Zia, Muti ur Rehman Khan, Usman Ali Ashfaq, Sanaullah Khan

**Affiliations:** 1 Molecular Parasitology and Virology Laboratory, Department of Zoology, Kohat University of Science and Technology, Kohat, Pakistan; 2 Department of Bioinformatics and Biotechnology, Government College University, Faisalabad, Pakistan; 3 Department of Biological Sciences, Forman Christian College, Ferozpur road, Lahore, Pakistan; 4 Department of Pathology, University of Veterinary and Animal Sciences, Lahore, Pakistan; 5 Department of Zoology, Kohat University of Science and Technology, Kohat, Khyber Pakhtunkhwa, Pakistan

**Keywords:** Rabies, Zoonosis, Vaccine, Prevention

## Abstract

Rabies is an avertable viral disease caused by the rabid animal to the warm blooded animals (zoonotic) especially human. Rabies occurs in more than 150 countries and territories. According to an estimation by WHO, almost 55,000 people die because of rabies every year. The Dogs are the major reason behind this, approximately 99% human deaths caused by dog's bites. Developing and under developing countries, both are the victims of rabies. With the post-exposure preventive regimes, 327,000 people can prevent this disease annually.

The current article mainly covers the genome, virology, symptoms, epidemiology, diagnostic methods, and the high risk countries around the globe.

## Background

Rabies is a zoonotic (transmitted from animals to human) viral infectious disease. This infection is transmitted to human by the animals already suffering from it. The animals which are mainly reported as causes of rabies are; dogs, raccoons, skunks, bats, and foxes. Rabies or "Hydrophobia" is a disease which makes the dogs sick. In many eastern and western countries dogs are vaccinated against it, but it is not controlled yet. Rabies is caused by a virus that, attacks on the nerves system and later excreted in saliva [1]. A person or animal can become a victim of rabies in many ways including [2],

a. Bites

b. Non-bites exposure

c. Human to Human Transmission

Bites from rabid animal to human are very common but the other two factors are rare [2]. Rabies affects the brain and spinal cord (central nervous system) with initial symptoms like; flu, fever, headache, but the infection can progress quickly to hallucinations, paralysis, and eventually death [3].

## Genome and virology

Rabies virus is the "type species" of the *Lyssavirus *genus of *Rhabdoviridae *family. The virus is enveloped and has a single stranded, negative sense RNA genome [4]. The RNA genome of the virus encodes five genes whose order is highly conserved. These genes codes for: nucleoprotein (N), phosphoprotein (P), matrix protein (M), glycoprotein (G), and a viral RNA polymerase (L). All rahbdoviruses have two major structural components; helical ribonucleoprotein core (RNP) and surrounding envelops. The two proteins, P and L are associated with RNP. The glycoprotein forms approximately 400 trimeric spikes, which are tightly arranged on the surface of the virus [5]. The virus nucleoprotein (N) plays critical role in replication and transcription. Both viral transcription and replication are reduced, if the nucleoprotein is not phosphorylated [6]. Rhabdoviruses cell surface receptors are not identified but some researches point outs the phospholipids, especially phophatidyl serine as the cell surface receptor molecule. After endocytosis, pH-dependent fusion with the membrane of the endocytic vesicle occurs. The polymerase which is carried out by the virus make five individual mRNA for each protein. These mRNAs are capped, methylated and polyadenylated. The polymerase then transcribes the negative-sense genomic RNA into positive sense strand. The switch between transcription and replication of genomic RNAs are controlled by the level of N protein [7].

## Epidemiology

### Rabies in Asia

Most of the developing countries in Asia are the victims of rabies. According to the WHO global vaccines research forum, over 30,000 people die every year due to rabies in Asia. One Asian dies every 15 minutes where 15% are likely to be the children under 15 years. More than 3 billion people in developing countries in Asia are exposed to dog rabies. According to WHO, high death rate was experienced in India in 2004 and lowest in Cambodia and Magnolia [8]. In 2006-2007, more than 3,000 rabies cases were reported which reduced to 205 in the year 2008 in China [9]. In India, about 15 million people are bitten by dogs every year and in 1985, it has been reported that 25,000-30,000 deaths are due to rabies annually, but due to preventive measures, the death rate reduced to 20,585 per year [10]. Nepal has one of the highest reported per capita rates of human rabies deaths in the world [11]. The Kathmandu Animal Treatment center claims that 200 people die in Nepal annually due to rabies as only in Kathmandu, 35,000 street dogs are reported. According to another research, there are 2,930 dogs per square kilometer in Kathmandu valley and the ratio between human and stray dogs is 4.7-1. And the huge population of stray dogs there increases the chances of rabies infection [12]. In Srilanka more than 95% of approximately 100 human rabies deaths occur each year due to the bites by unvaccinated dogs [13]. Rabies is an important public health problem in Bangladesh also where nearly 100,000 people being bitten by dogs in 2009 and 3,000 died of rabies [14]. Pakistan is also facing a threatening situation regarding rabies. Only in Karachi, the estimated population having rabies is 9 per million [15]. Everyday there are about 25-30 new cases of dog-bites treated by the doctors at civil hospital Karachi, one of the biggest public hospitals in city. According to a study by the National Rabies control program Pakistan, Punjab, Sindh and the Khyber Pakhtunkhwa as well as few districts of Naseerabad, Jafferabad, and Pishin in Baluchistan are categorized as the high risk areas for rabies [16].

### Rabies in Africa

Rabies causes at least 24,000 deaths per year in Africa. The high death rates reported in poor rural communities and children [17]. The major cause of spread of rabies in this region is urbanization [18]. An unconfirmed epidemic of rabies in dogs occurred in western Zambia in 1901. The existence of rabies was confirmed in South Africa in 1928 [19]. Other southern African countries like; Angola, Namibia, Mozambique, Zimbabwe are also considered as high risk areas.

### Rabies in Europe and united states

Rabies is still present in Europe, but the human rabies has been disappeared from many European countries (Table 1). The disappearance of rabies was probably due to enforced policy of animal vaccination. The epidemiological and genetic analysis of many isolates showed that canine rabies remains in certain countries as well as borders of Europe [20]. In 1946, a total of 8,384 indigenous rabies cases were reported among dogs and 33 cases in human in USA. After that there was noticeable decrease in rabies cases in USA. In 2006, none of human rabies case was reported in the country [21].

**Table 1:** Rabies free countries and territories

Asia	America	Europe	Oceana	Africa
Bahrain	Antigua and Barmuda	Albania	Cook Islands	Cape Verde

Cyprus	Bahamas	E.Y.R. of Macedona	Fiji	Congo

Hong Kong	Barbados	Finland	French Polynesia	Libya

Japan	Belize	Gibraltar	Guam	Mauritius

Malaysia	Falkland	Greece	New Caledonia	Reunion

Maldives	Jamaica	Iceland	New Zealand	Seychelles

Qatar	Saintkitts and Nevis	Isle of Man	Papua New Guinea	

Singapore	Trinidad and Tobago	Malta	Solomon Islands	

Lakchyadeep, Andaman and Nicobar islands of India	Uruguay	Norway (except Svalbard and isl.)	Vanuatu	

Timor-Leste		Portugal		

		Spain (except centa + Melill)		

		United Kingdom		

### Pathogenesis

The common mode of transmission of rabies in man is by bite of a rabid animal or the contamination of scratch wounds by virus infected saliva. Rabies is an acute infection of the central nerves system (CNS) which is almost invariably fatal. Following inoculation, the virus replicates in the striated or connective tissue at the site of inoculation and enters the peripheral nerves through the neuromuscular junction. It then spreads to the CNS in the endoneurium of the Schwann cells. Terminally, there is widespread CNS involvement but few neurons infected with the virus show structural abnormalities. The nature of the profound disorder is still not understood [22].

## Diagnosis of rabies virus

### Diagnosis in animals

The diagnosis of rabies in animals can be made by taking any part from the affected brain. But in order to rule out rabies, the test must include tissues from at least two locations in brain, from the brain stem and cerebellum [23]. There are many diagnosis methods for detection of rabies in animals like (Table 2); direct florescent antibody, mouse inoculation technique, tissue culture infection technique, and polymerase chain reaction. All these techniques are recommended by WHO [24].

**Table 2:** Diagnosis techniques for rabies

Technique	Specimen	Advantage/Disadvantage
Direct Fluorescent Antibody Technique (DFA)	Target organs, such as brain, salivary glands, liver, spleen, pancreas, nuchal skin, brain is the most appropriate sample	Applicable with most tissue sources. Not applicable in decomposed tissue

Mouse Inoculation Technique (MIT)	Similar to DFA	Only use fresh tissues

Tissue Culture Infection technique (TCIT)	Similar to DFA	Only use fresh tissues

Polymerase Chain Reaction (PCR)	Similar to DFA including body fluids, saliva, urine, CSF	Applicable in all tissue conditions Expensive Need experienced technicians

### Clinical diagnosis in human

Clinical diagnosis of rabies divided upon three stages; prodromal, excitement (furious) and paralytic (dumb). But all these stages cannot be observed in an individual [25]. The very first clinical symptom is neuropathic pain at the site of infection or wound due to viral replication. Following by the prodromal phase either or both the excitement or paralytic forms of the disease may be observed in the particular species. It is also documented that cats are more likely to develop furious rabies than dogs [26]. In some cases, no signs are observed and rabies virus has been identified as the case of sudden death [27]. Diagnosis can only be confirmed by laboratory tests preferably conducted post mortem on central nervous system tissue removed from cranium [28]. Tests are also performed on the samples of saliva, serum, and skin biopsies of hair follicles at the nape of the neck [23].

## Prevention and treatment

### Vaccine

There is no certain cure for rabies except supportive care. Rabies can be prevented before the latent symptoms can develop, consists of giving a person an injection of rabies immune globulin and another injection of rabies vaccine as soon as possible after the bite or exposure to saliva from an infected animal. Human rabies immune globulin is used or injected at the bite area immediately because it attacks the virus and slow down or stop viral progression through the nerves [29]. Timing and the ability of the patient to respond by making a good immune response is a key to patient survival. Untreated or inappropriately treated rabies is almost always fatal because treatment is supportive only to limit the patient's pain. An effective new rabies treatment regime that gives the protection from the disease is developed by the scientists. The treatment regimes are; Post-exposure prophylaxis and Pre-exposure prophylaxis.

### Post-exposure prophylaxis

If a person is bitten by an animal, the wound and scratches should be washed thoroughly with soap and water to decrease the chances of infection. Post-exposure prophylaxis involved one dose of rabies immune globulin and five doses of rabies vaccine within the 28 days period. Rabies immune globulin contains antibodies from blood donors who were given rabies vaccine. The rabies vaccine works by stimulating a person's immune system to produce antibodies that neutralize the virus.

### Pre-exposure prophylaxis

The people who are considered as high risk group need pre-exposure prophylaxis. These groups includes; a-veterinarian, animal handlers and laboratory workers; b-the people whose activities bring them in contact with rabies virus or rabid animals; c-international travelers likely to come in contact of the animals in the rabies threaten areas. All these groups should be treated with rabies vaccines to avoid the chances of sudden infection.

### Common issues and concerns about rabies

There are certain issues which should be addressed to prevent this fatal disease. Rabies is not considered as a priority disease in most of the countries, especially in Asia. Secondly, there is insufficient surveillance system in developing countries. Mostly in developing countries, there is limited access to modern rabies vaccines and immunoglobulin. The most burning issue is lack of awareness among the common people in high-risk countries. If these concerns can be settled down properly, the prevention from rabies is possible.

### Recommendations

Rabies is a fatal viral zoonotic disease and a dangerous public health problem. Mostly European countries are totally saved from this disease by practicing prevention measures. Public health education workshops should be organized to educate people about responsible pet ownership and routine veterinary care. The majority of animal and human exposures to rabies can be prevented by raising awareness concerning: rabies transmission routes, avoiding contact with wildlife, and following appropriate veterinary care. Human rabies prevention is most important and can be prevented either by eliminating exposures to rabid animals or by providing exposed persons with prompt local treatment of wounds combined with the administration of human rabies immune globulin and vaccine. Local governments should initiate and maintain effective programs to ensure vaccination of all dogs, cats, and ferrets and to remove strays and unwanted animals.

## Conclusion

Rabies is a viral disease that can be spread by domestic and wild animals. Many countries having the status of high-risk areas but most of the countries around the globe gained the status of rabies free territories. This shows that rabies can be successfully ruled out from the high-risk areas by taking preventing measures. The advent of scientific medicine also makes rabies control possible. Public awareness in this regard can play major role. There is needed to take little care and change of lifestyle to avoid these kinds of viral diseases.

## Abbreviations

WHO: World Health Organization; CNS: Central Nervous System.

## Competing interests

The authors declare that they have no competing interests.

## Authors' contributions

MZY conceived the study and drafted the manuscript. MQ equally contributed in final drafting of the manuscript. SZ and MRK searched the literature and helped in manuscript write-up. UAA and SK critically review the manuscript. All authors read and approved the final manuscript.

## Authors' information

Muhammad Z Yousaf (PhD Molecular Biology), Muhammad Qasim (PhD Molecular Biology), Muti ur Rehman Khan (PhD Molecular Biology), Usman A Ashfaq (PhD Molecular Biology), Sanaullah Khan (PhD Scholar), Sadia Zia (M-Phil Scholar)

## References

[B1] What is rabies? http://www.netdoctor.co.uk/travel/diseases/rabies.htm

[B2] What Causes Rabies? An Introduction http://rabies.emedtv.com/rabies/what-causes-rabies.html

[B3] What is rabies? http://www.bettermedicine.com/article/rabies-1

[B4] Drew WL Ryan KJ, Ray CG Rabies Sherris Medical Microbiology 2004 4 McGraw Hill 597 600

[B5] The rabies virus http://www.cdc.gov/rabies/transmission/virus.html

[B6] Wu Xianfu Gong Xiaoming Heather D Foley Matthias J Schnell Fu ZF Both viral transcription and replication are reduced when the rabies virus nucleoprotein is not phosphorylated J Virol 2002 76 9 4153 4161 10.1128/JVI.76.9.4153-4161.2002 11932380 PMC155083

[B7] Virology http://pathmicro.med.sc.edu/virol/rabies.htm

[B8] Raux H Flamand A Blondel D Interaction of the rabies virus P protein with the LC8 dynein light chain J Virol 2000 74 10212 10216 10.1128/JVI.74.21.10212-10216.2000 11024151 PMC102061

[B9] Iwasaki Y Tobita M Jackson AC, Wunner WH Pathology Rabies 2002 San Diego: Academic 283 306

[B10] The Path of the Virus http://www.cdc.gov/rabies/transmission/body.html

[B11] WHO global vaccine research forum: Epidemiology of rabies in Asia http://www.who.int/entity/vaccine_research/about/gvrf/en/index.html

[B12] in China http://www.rabiesinasia.org/china-wrd2008.html

[B13] Rozario M Rabies in India CMAJ 2008 178 5 564 566 10.1503/cmaj.071488 18299543 PMC2244675

[B14] Rabies Control in Nepal http://www.tufts.edu/vet/deph/ivm/projects/rabies_control.html

[B15] Rabies In Nepal And Its Prevention http://www.gorkhapatra.org.np/rising.detail.php?article_id=40201&cat_id=7

[B16] Smith JS Rupprecht CE Rabies in Sri Lanka: splendid isolation Susilakanthi Nanayakkara Emerging Infectious Diseases 2008 9 03 10.3201/eid0903.020545 PMC2958551 12643834

[B17] Rabies emerging as major killer in Bangladesh http://www.weeklyblitz.net/904/rabies-emerging-as-major-killer-in-bangladesh

[B18] Niloufer SA Guidelines for prophylaxis of rabies in Pakistan Pak J Med Sci 2003 19 1 61 65

[B19] Pakistan: Effective surveillance of rabies imperative http://www.irinnews.org/report.aspx?reportid=26491

[B20] Rabies in Africa http://www.sciencedaily.com/releases/2009/01/090121091237.htm

[B21] Evolutionary history and dynamics of dog rabies virus in western and central Africa http://www.sgm.ac.uk/JGVDirect/007765/007765ft.pdf 10.1099/vir.0.007765-0 19264663

[B22] Pathogenesis of rabies infection http://virology-online.com/viruses/Rhabdoviruses3.htm

[B23] Swanepoel R Barnard BJ Meredith CD Bishop GC Brückner GK Foggin CM Hübschle OJ Rabies in Southern Africa J Vet Res 1993 60 4 325 46 7777317

[B24] Bourhy H Dacheux L Strady C Mailles A Rabies in Europe in 2005 Euro Surveillance 2005 10 11 10.2807/esm.10.11.00575-en 29208125

[B25] Recommendations of the Advisory Committee on Immunization Practices http://www.cdc.gov/mmwr/preview/mmwrhtml/rr5703a1.htm

[B26] Diagnosis in animals & humans http://www.cdc.gov/rabies/diagnosis/animals-humans.html

[B27] Boonlert L Laboratory Techniques for Rabies Diagnosis in Animals at QSMI Thai Red Cross Society J Med Assoc Thai 2005 88 4 550 553 16146265

[B28] McElhinney M Fooks AR Radford AD Diagnostic tools for the detection of rabies virus EJCAP 2008 18 03

[B29] Fogelman V Fischman HR Horman JT Grigor JK Epidemiologic and clinical characteristics of rabies in cats J Am Vet Med Assoc 1993 202 1829 1838 8320150

